# Gadd45β expression in chondrosarcoma: A pilot study for diagnostic and biological implications in histological grading

**DOI:** 10.1186/1746-1596-5-69

**Published:** 2010-10-13

**Authors:** Michihisa Zenmyo, Akihide Tanimoto, Harutoshi Sakakima, Masahiro Yokouchi, Satoshi Nagano, Takuya Yamamoto, Yasuhiro Ishido, Setsuro Komiya, Kosei Ijiri

**Affiliations:** 1 Orthopaedic Surgery, Graduate School of Medical and Dental Sciences, Kagoshima University, Kagoshima, Japan; 2 Molecular and Cellular Pathology, Graduate School of Medical and Dental Sciences, Kagoshima University, Kagoshima, Japan; 3 School of Health Sciences, Faculty of Medicine, Kagoshima University, Kagoshima, Japan

## Abstract

**Background:**

Although the diagnosis of chondrosarcoma, especially the distinction between enchondroma and low-grade chondrosarcoma or low-grade chondrosarcoma and high-grade chondrosarcoma, is pathologically difficult, differential diagnosis is very important because the treatment strategies for these diseases are completely different. The grading system is crucial in predicting biologic behavior and prognosis, however, exact pathological grading is difficult using only routine examinations because the criteria of the grading system are not necessarily definitive. Growth arrest and DNA damage-inducible protein 45β (GADD45β) is an essential molecule for chondrocytes during terminal differentiation. In the present study, we investigated the immunohistochemical expression of GADD45β in enchondroma, and chondrosarcoma of histological grades I, II, and III, to clarify the diagnostic significance of GADD45β in pathological grading of chondrosarcoma.

**Methods:**

Twenty samples (enchondroma = 6, chondrosarcoma grade I = 7, grade II = 6, grade III = 1) were used for immunohistochemical analysis to investigate the expression of GADD45β. Quantitative analysis was performed to compare the number of GADD45β positive cells and pathological grading.

**Results:**

Over 70% of the cells in enchondromas expressed GADD45β. On the other hand, the expression of GADD45β decreased significantly according to the histological grade of chondrosarcoma (grade I: 45%; grade II: 13.8%; and grade III: 3.8%).

**Conclusions:**

The association of GADD45β expression and pathological grading of chondrosarcoma in the present study suggests that the immunohistochemical study of GADD45β may be a specific diagnostic parameter for chondrosarcoma cell differentiation.

## Background

Chondrosarcoma is the second most frequent primary malignant bone tumor [1,2]. Because of its recalcitrance to chemotherapy and radiotherapy, chondrosarcoma is primarily treated with surgery, and the clinical prognosis of chondrosarcoma has been correlated with the grading of the histological malignancy [3].

For pathological consideration, distinguishing benign (enchondroma) from low-grade chondrosarcoma, or low-grade chondrosarcoma from high-grade chondrosarcoma, is one of the most frequent diagnostic dilemmas facing orthopedic oncologists.

Enchondroma is a very common and benign cartilaginous tissue tumor that occurs within bones. Approximately 69% of the patients are in the first and second decades of life [4]. More than 49% of the tumors are in the small bones of the hands and feet, particularly in the phalanges. Unlike chondrosarcoma, enchondroma rarely develops in the pelvis or ribs. The pathological distinction between enchondroma and low-grade chondrosarcoma is, however, not always easy because of their similar cytology and cellularity. Chondrosarcoma has a broad array of presentations in pathology and clinical course. Chondrosarcoma is primarily a tumor of adulthood and old age. Approximately 62% of the patients are in the fourth to sixth decades. More than two-thirds of the tumors are in the trunk, including the pelvis, ribs, and shoulder girdle. The pathological grading of chondrosarcoma is based on cellularity, nuclear atypia, and pleomorphism [5]; however, in some borderline cases, exact histological grading is difficult using only routine histopathological examinations because the criteria of the grading system are not necessarily definitive [6]. Therefore, correlative interpretation of histopathological, imaging, and clinical information is currently used for making this distinction.

Several authors have reported supplementary methods, including the evaluation of DNA synthesis and content [7,8], flow cytometry[9], p53 [10], MIB-1 [11], COX-2 [6], and p21 [3], to assess the prognosis of patients with chondrosarcoma. These methods are, however, based on non-specific phenomena in chondrocytic differentiation.

Chondrogenesis, i.e., cartilage formation including chondrocyte differentiation and maturation, is a process that occurs during skeletal development. This process occurs in stages beginning with mesenchymal cell recruitment and migration, proliferation, and condensation, followed by chondroprogenitor cell determination and differentiation. Finally, chondrocyte differentiation is terminated by hypertrophy. Bone morphogenetic proteins (BMPs), which were originally identified as molecules that induce ectopic endochondral ossification [12], set the stage for bone morphogenesis by initiating chondroprogenitor cell determination and differentiation and regulate the later stages of chondrocyte maturation and hypertrophic phenotype [13].

We previously reported the growth arrest and DNA damage-inducible protein 45β (GADD45β) as an early responding gene to BMP-2 stimulation in the chondrocyte cell line [14]. The expression of GADD45β gradually increased along with chondrocyte differentiation from the proliferation phase to hypertrophic phase. GADD45β stimulates MMP-13 (a marker of terminal differentiation of hypertrophic chondrocytes) promoter activity in chondrocytes through the JNK-mediated phosphorylation of JunD, partnered with Fra2 and in synergy with Runx2. These facts suggested that GADD45β plays an essential role during chondrocyte terminal differentiation.

In the present study, we investigated the immunohistochemical expression of GADD45β in enchondroma and chondrosarcoma of histological grades I, II, and III, to clarify the diagnostic significance of GADD45β in histological grading of chondrosarcoma.

## Methods

### Study samples

Formalin-fixed, paraffin-embedded tissues collected between 1978 and 2009 were obtained from the Department of Pathology, Graduate School of Medical and Dental Sciences, Kagoshima University upon approval of the ethics committee. The characteristics of the patients are summarized in Table 1.

**Table 1:** Patient characteristics

Case	Sex/Age	Location	Pathology & Grade	GADD45b positivity (%)
1	♂/43	Femur	Enchondroma	68.9
2	♂/16	Metatarsal	Enchondroma	81.6
3	♀/46	Phalanx	Enchondroma	74.4
4	♂/32	Phalanx	Enchondroma	83.5
5	♀/58	Femur	Enchondroma	66.6
6	♀/54	Phalanx	Enchondroma	68.8
7	♀/52	Femur	CSA grade I	67.3
8	♂/39	Pelvis	CSA grade I	55.5
9	♀/46	Femur	CSA grade I	44.6
10	♂/61	Femur	CSA grade I	49.2
11	♂/18	Tibia	CSA grade I	29.8
12	♂/67	Mandible	CSA grade I	38.1
13	♀/32	Pelvis	CSA grade I	31.0
14	♀/50	Femur	CSA grade II	11.8
15	♀/64	Rib	CSA grade II	3.9
16	♀/72	Femur	CSA grade II	14.8
17	♀/55	Pelvis	CSA grade II	5.9
18	♂/44	Femur	CSA grade II	11.9
19	♂/67	Mandible	CSA grade II	34.0
20	♂/61	Mandible	CSA grade III	3.8

### Pathological review and grading

Histological slides of the tumors from all the patients were reviewed by 2 or 3 pathologists. The histological diagnosis was based on the textbook definition [15]. The histological grade of the chondrosarcomas was determined on the basis of the nuclear size, nuclear staining (hyperchromasia), and cellularity according to the World Health Organization Classification of Bone Tumors (2002) [15].

### Immunohistochemical analysis

The immunohistochemical analysis was performed using the labeled streptavidin-biotin method using goat polyclonal anti-human GADD45β (C-18; 1:1000) antibody (Santa Cruz Biotechnology, Inc. CA, USA). The results were evaluated by 2 investigators, who were unaware of the pathological grading of the samples. We calculated the proportion of stained tumor cells after performing nuclear staining.

Quantitative analysis of the GADD45β immunoreactive cells was performed. The immunostained sections were photographed at 100× magnification with a microscope, and the GADD45β immunoreactive cells and non-positive cells were counted in 4 fields. We calculated the percentages of GADD45β positive cells. Statistical comparisons in each grade (enchondroma, grade I and II) were performed by a one-way ANOVA using Stat-View version 5.0. If significance was achieved, a post-hoc Fisher's protected least significant differences (PLSD) test was performed to determine whether significant differences existed. Significance was set at p < 0.05.

## Results

**Figure 1:**
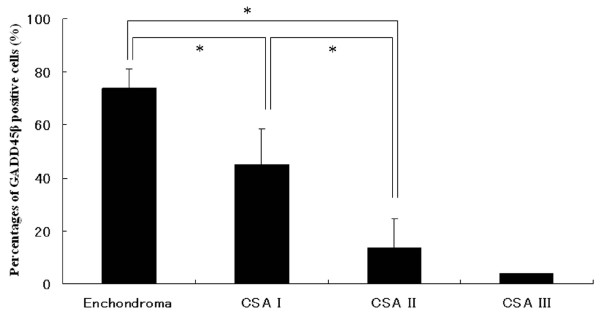
**Percentages of GADD45β positive cells**.

The patient data and lesion characteristics are listed in Table 1. There were 6 cases of enchondroma and 14 of chondrosarcoma, including 7 with grade I, 6 with grade II, and 1 with grade III. Enchondromas, on average, showed 74% reactivity for GADD45β (68.9-83.5%). In the case of chondrosarcomas, the percentage of GADD45β positive cells was inversely proportional to the tumor grade. GADD45β was expressed in 45% (29.8-67.3%) of the cells in grade I chondrosarcoma and 13.8% (1.2-34.1%) of cells in grade II. There were statistically significant differences among the three groups except grade III chondrosarcoma. (Fig. 1)

**Figure 2:**
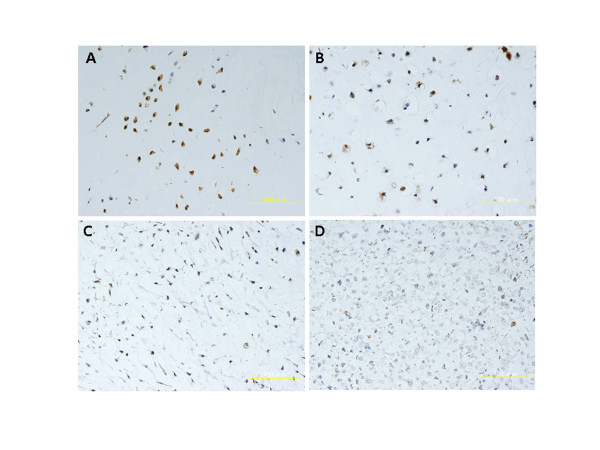
**Immunohistochemical staining for GADD45β A) Enchondroma, B) Grade I Chondrosarcoma, C) Grade II Chondrosarcoma, D) Grade III Chondrosarcoma**. Chondrocytes which have plump nuclei and are surrounded by cartilage matrix, are positive for GADD45β. In contrast, most of the atypical and immature cells in grade II or III chondrosarcoma are negative. Over 70% of the cells in enchondromas expressed GADD45β. In chondrosarcomas, the rate of GADD45β positive cells was inversely proportional to its grade. There were statistically significant differences among three groups except grade III chondrosarcoma. (*: P < 0.01)

In grade II chondrosarcomas, comparatively mature cells, which had plump nuclei and were surrounded by cartilage matrix, were positive for GADD45β. In contrast, most of the atypical spindle cells, which had enlarged hyperchromatic nuclei and myxomatous matrix, were negative. (Fig. 2C) Although we had only 1 case of grade III chondrosarcoma, only 3.8% of the cells were positive for GADD45β.

## Discussion

The pathological grade of chondrosarcomas is considered to be the most useful predictor of the clinical outcome and course of the treatment for these tumors. While criteria for pathological grade of chondrosarcomas have been published, the application of these criteria is difficult and requires expert judgement [16].

Therefore, correlative interpretation of histopathological, imaging, and clinical information is currently used as a method to provide the appropriate prognostic information. Recently, the reliability of histopathological and radiological grading of cartilaginous neoplasms in long bones was studied by 9 recognized musculoskeletal pathologists and 8 recognized musculoskeletal radiologists [17]. They reviewed 49 consecutive cases of cartilaginous lesions in long bones and estimated the interobserver reliability for the grading of cartilaginous neoplasms in long bones to be 0.443 for pathologists and 0.345 for radiologists. These data demonstrate that the interpretation of histopathological features from cartilaginous lesions has some limits. These data encouraged the efforts to develop better prospective pathological interpretation algorithms or better prospective imaging protocols.

The present study provides the first immunohistochemical evidence that GADD45β expression is significantly associated with the grading of chondrosarcomas. GADD45β is a member of the GADD45 family of small (18 kDa) proteins, which are known to be associated with cell growth control, apoptotic cell death, and the cellular response to DNA damage [18]. Initially, GADD45β encoded by *MyD118 *was identified as a myeloid differentiation primary response gene activated by IL-6 in murine myeloid leukemia cells upon induction of terminal differentiation [19].

Formation of the vertebral skeleton through endochondral ossification is a physiological process, which involves progressive differentiation of proliferating chondrocytes to growth-arrested hypertrophic chondrocytes. Interestingly, the GADD45β protein localized prominently in the nucleus of the late-stage hypertrophic chondrocytes, but not of the proliferating chondrocytes. Although GADD45β is an abundant protein in chondrocytes, overexpression of GADD45β in terminally differentiated chondrocytes in the growth plate promotes JNK and p38 signaling cascades to regulate both MMP-13 and Col10a1 expression, which are crucial for the maintenance of the terminal differentiated phenotype [20]. Therefore, the association of GADD45β expression and pathological grading of chondrosarcoma in the present study suggests that immunohistochemical study of GADD45β may be a specific diagnostic parameter for chondrosarcoma cell differentiation.

In this study, there was overlap in the proportion of GADD45β-expressing cells between enchondroma and grade I chondrosarcoma (CSA), as shown for Case 7, and between grade I CSA and grade II CSA, as shown for Case 19. The limitation of this pilot study of GADD45β expression in chondrogenic tumors was the small number of subjects. Therefore, the study should be conducted on a larger number of subjects to determine whether the number of such "overlap" cases would increase.

## Conclusions

In conclusion, our data suggests that immunohistochemical study of GADD45β provides valuable diagnostic information and facilitates examination of histological grading. More studies involving larger number of patients must be performed to further characterize its potential diagnostic validity.

## Competing interests

The authors declare that they have no competing interests.

## Authors' contributions

ZM, AT, MY, SN carried out the immunohistochemical studies. TY, YI participated in the design of the study, and SH contributed in the statistical analysis. SK and KI conceived the study and drafted the manuscript. All authors read and approved the final manuscript.
